# The Production of Clitics in Serbian Speakers with Stroke Aphasia

**DOI:** 10.3390/brainsci16030324

**Published:** 2026-03-19

**Authors:** Mile Vukovic, Sladjana Lukic

**Affiliations:** 1 Faculty of Special Education and Rehabilitation, University of Belgrade, 11000 Belgrade, Serbia; mvukovic@fasper.bg.ac.rs; 2 The Neurobiology of Language and Behavior Laboratory, School of Communication Science and Disorders, College of Communication and Information, Florida State University, Warren Building, 201 W Bloxham St., Tallahassee, FL 32301, USA

**Keywords:** clitic production, stroke aphasia, Serbian morphosyntax

## Abstract

**Highlights:**

**What are the main findings?**

- Nonfluent aphasia was associated with impaired production of both enclitics and proclitics, whereas fluent aphasia showed a selective advantage for proclitics.
- Clitic production was differentially related to working memory and sentence repetition across clitic and aphasia types, with broader associations in nonfluent aphasia.

**What are the implications of the main findings?**

- Serbian clitic production may provide a sensitive linguistic marker for distinguishing aphasia profiles.
- Interventions targeting sentence-level planning and working memory may enhance clitic production, particularly in nonfluent aphasia.

**Abstract:**

**Background/Objectives:** Cross-linguistic studies show that the production of morphosyntactic elements (e.g., clitics) is problematic and often omitted in nonfluent agrammatic aphasia (NFA), with the degree of impairment varying across languages. Serbian, with its rich clitic system, provides a sensitive window into grammatical impairment. This study is the first to examine the production of proclitics and enclitics in Serbian speakers with aphasia and their relationship to short-term and working memory. **Methods**: Forty-six individuals with stroke-induced aphasia (25 NFA and 21 fluent aphasia [FA]) and 54 healthy controls completed an experimental Serbian clitic production test. Participants were prompted to produce clitic sentences (12 proclitics, such as prepositions or conjunctions, and 18 clitics, such as pronouns or auxiliary verbs) in response to various scenarios. Performances were correlated with sentence repetition and digit span (forward/backward). **Results**: Both aphasia groups produced significantly fewer clitics than controls (*p* < 0.001). Participants with NFA produced fewer overall clitics and showed no clitic type effects (*p* = 0.329), whereas participants with FA produced proclitics more accurately than enclitics (*p* = 0.028). Clitic production correlated with performance on sentence repetition and digit span tasks, but patterns differed by aphasia group. In NFA, both enclitics and proclitics were associated with sentence repetition and digit span (*p* < 0.05), whereas in FA, these measures were primarily associated with enclitic production (*p* < 0.05). **Conclusions**: Clitics production distinguishes nonfluent from fluent aphasia in Serbian and is differentially supported by working and verbal memory resources. The Serbian clitic production test reveals a selective proclitic advantage that is observed only in fluent aphasia, serving as a sensitive clinical marker in this population.

## 1. Introduction

Clitics (e.g., pronouns, prepositions) are morphosyntactic elements that lack prosodic independence and full lexical status, derived through syntactic movement and obligatorily attached to a host word [1,2]. Cross-linguistic research reveals substantial variation in the distribution, morphosyntactic behavior, and interpretation of clitics, resulting in diverse classification criteria across languages [3,4,5,6]. For example, Italian clitics are classified using functional criteria [7] into determiners, prepositions, and pronouns [4], whereas in French, nearly all personal pronouns (except disjunctive forms) are clitics with a strictly fixed order [8,9]. In Spanish, clitics are weak personal pronouns that must attach to a verbal host, while in English, pronouns have sometimes been analyzed as clitic-like mainly because of their accentless realization.

Serbian is of particular interest because it has one of the most complex clitic systems among Slavic languages. Clitics in Serbian play a central grammatical role, marking tense, aspect, person, and syntactic relations [10,11,12]. This makes them especially sensitive indicators of grammatical competence and potential markers of language impairment in aphasia. Speakers alternate between unstressed (clitic) and stressed (full) forms depending on syntactic and prosodic context. For instance, clitic forms are preferred in contexts like ‘Gledala *ga* je netremice.’ (“She looked at *him* without blinking.”), whereas full forms may be chosen in ‘Bilo kako bilo, *njega* je prepoznala.’ (“Anyway, she did recognize *him*.”). Syntactically, they are considered syntactic constituents, typically functioning as functional words. Clitics may cluster together but must appear in a fixed order (e.g., ‘*Taj* mi je pesnik napisao pesmu.’—“*That* poet wrote a poem to me.”). Word order variation does not affect clitic sequence (e.g., ‘*Taj* pesnik mi je napisao pesmu.’—“*That* poet wrote me a poem.”) [4].

A central distinction in Serbian is between enclitics and proclitics, defined by their position relative to the host word. *Enclitics* are dependent elements that attach to the preceding word and cannot occur sentence-initially or sentence-finally, despite the relative flexibility of Serbian word order [10]. They include unstressed pronouns (genitive, accusative, and dative forms), auxiliaries, and reduced forms of the verbs *to be* (*je*) and *to want* (*će*) [10,13,14]. Auxiliary clitics *je* and *će* differ in grammatical function, temporal reference, and morphosyntactic behavior, and may co-occur with object pronoun clitics in complex constructions that are particularly demanding for grammatically impaired speakers (e.g., *Popila ga je mačka* “The cat drank it”). In contrast, *proclitics*, such as prepositions or conjunctions, attach to the following word and may occur sentence-initially (e.g., *Učenici sede na drvenim stolicama* “Students sit on wooden chairs”) [11,12].

Deficits in clitic production are well documented in individuals with aphasia, particularly those with Broca’s agrammatic nonfluent aphasia, and have been reported across Romance languages. Compared to neurologically healthy individuals, Italian speakers with agrammatic aphasia produced fewer pronominal clitics [15,16,17] as well as fewer reflexive, direct object, and indirect object clitics [18]. Reznik et al. [19] reported that 56% of clitics were omitted in Spanish speakers with agrammatic aphasia, particularly object and reflexive clitics [20,21,22,23,24], and have been reported across Spanish, Catalan, and Galician [25]. Greek-speaking individuals with agrammatic aphasia exhibited greater impairment in the use of direct object clitics, while personal pronouns and genitive clitics were relatively preserved [26,27,28]. Finally, reduced use of demonstratives, possessives, personal and relative pronouns, prepositions, and subordinate conjunctions has also been reported in two Serbian-speaking individuals with agrammatic aphasia during narrative production [29,30,31].

Despite extensive cross-linguistic research (see [32] for a review), no study has systematically examined clitic production in Serbian speakers with aphasia, creating a basis for identifying novel clinical insights. This gap can be addressed by contrasting different clitic types (enclitic and proclitic) in nonfluent and fluent aphasias (NFA and FA). Preliminary findings already suggest significant clitic deficits in adults with NFA, particularly affecting enclitics such as the reflexive pronoun *se* and auxiliary verbs *jesam* during spontaneous speech [33]. Together, these findings highlight the diagnostic potential of Serbian clitic production tasks for identifying grammatical impairments in individuals with aphasia. Importantly, clitic production may also relate to broader cognitive functions. Language and memory are closely linked, and deficits in short-term and working memory are common in aphasia [34,35,36]. To address these issues, we developed a Serbian Clitic Production (SCP) Test targeting language-specific grammatical features, including tense, aspect, question formation, and pronouns [37]. We hypothesized that individuals with aphasia (specifically, NFA) would produce significantly fewer clitics than neurologically healthy controls, and that reduced clitic production would be associated with reduced working memory capacity.

## 2. Materials and Methods

### 2.1. Participants

The study included 54 right-handed healthy controls (33 females, age range = 21–83, M (SD)= 52.93 (17.97)) and 46 right-handed individuals with aphasia (14 females, age range = 18–74, M (SD)= 59.28 (10.54)). Participants with aphasia were at least 6 months post-onset of stroke (6–18 months, M (SD) = 14.41 (5.2)) and included: 17 individuals with Broca’s aphasia, 5 with Wernicke’s aphasia, 7 with Anomic aphasia, 7 with Conduction aphasia, 8 with Transcortical Motor Aphasia (TMA), and 2 with Transcortical Sensory Aphasia (TSA). Participants with aphasia were recruited from a hospital outpatient Rehabilitation Clinic in Serbia and were classified into one of the types of aphasia based on the Boston Diagnostic Aphasia Examination (BDAE-2) [38], which was translated and adapted to Serbian [39]. Inclusion criteria were (1) monolingual native speakers of Serbian language, (2) right-handed, (3) at least 8 years of education and 18 years of age, (4) stroke-induced aphasia, resulting from a single left hemisphere ischemic or hemorrhagic event, (5) no history of neurodevelopmental/neurodegenerative or psychiatric disorder, and (6) adequate pure-tone audiometric screening and normal or corrected-to-normal vision (self-reported).

The aphasia group was further differentiated into two broad categories: 25 nonfluent (Broca’s aphasia, TMA) and 21 fluent (Wernicke’s aphasia, Anomic aphasia, Conduction aphasia, TSA), given an unequal number of participants within each aphasia type. The study was approved by the Ethics Committee at the Clinic for Rehabilitation, Dr. Miroslav Zotović, Belgrade, Serbia. All participants voluntarily agreed to participate in the study and provided written consent in accordance with the Helsinki Declaration. A group of 54 healthy controls voluntarily agreed to participate in the study and provided informed consent, and their cognitive and speech-language abilities were assessed. Demographics, cognitive scores, speech-language scores, and expected significant group differences are provided in Table 1.

### 2.2. Materials and Procedure

#### 2.2.1. Serbian Clitic Production (SCP) Test

All participants completed an experimental Serbian Clitic Production (SCP) test [37]. The SCP test consists of 18 enclitics and 12 proclitics (total *n* = 30). Enclitics targets included reflexive pronouns (*se* “herself”), object pronouns (*ga* “him”), present-tense auxiliary verbs (*je* “to be”), and future-tense auxiliary verbs (*će* “to want”). Proclitic targets included prepositions (*iz* “from”) and conjunctions (*ili* “or”).

Participants were prompted to produce clitic-containing sentences in response to brief discourse scenarios designed to elicit specific target clitic(s) (see Table 2 for examples; the full list of stimuli is provided in the Appendix A). Word frequency was computed for each word and averaged within sentences to yield a sentence-level frequency measure using the Serbian Frequency Database by Lukić, V. (1983). Mean sentence-level frequencies were then compared between enclitic (*n* = 18) and proclitic (*n* = 12) conditions using Welch’s independent-samples *t*-tests. The two clitic conditions were matched in frequency of occurrence at the scenario level (*p* = 0.821) and the response level (*p* = 0.791).

Findings on healthy individuals indicate that the SCP demonstrated strong psychometric properties, including internal consistency, test–retest reliability, and both intra- and inter-rater reliability, and also showed good construct validity [37].

#### 2.2.2. Procedure

Participants are presented with a situation described in one or two sentences, followed by a question. They are required to produce an answer that, in natural speech, makes use of clitics. For each item, participants are presented with a prompt and are required to produce the appropriate clitic(s) based on the text. One point is awarded for each correctly produced clitic. For both enclitics and proclitics, responses—including self-corrections—were scored as correct if produced within 10 s. Some items (*n* = 14) require the production of two clitic types (e.g., *ga je* pronoun auxiliary). In these cases, 1 point is awarded only if both clitics are produced correctly; 0.5 points are given for producing only one clitic. Therefore, participants could score a total of either 18 or 12 points depending on the type of clitic tested. A clitic performance score was calculated for each participant as the percentage of correctly produced clitics for enclitics and proclitics, based on the number of correct responses relative to the total possible score. This performance score was used to assess differences in clitic production across clitic types and aphasia profiles. Prior to the actual test, participants received detailed instructions and completed two practice items with feedback to familiarize themselves with the procedure. All testing was administered by trained examiners (the first author and the speech-language pathologist assistant) following a standardized protocol to minimize variability in delivery and assistance.

#### 2.2.3. Cognitive Measures

To evaluate the relationship between clitics and higher-order cognitive abilities, we selected two standardized measures of cognitive functions from our comprehensive battery that relate to short-term memory (as measured by the digit forward and sentence repetition test) and working memory (as measured by the digit backward span task).

For the digit span, participants read increasingly longer sequences of numbers and were asked to repeat them immediately in the order presented. The digit spans were randomly generated with the restriction that no digit would be adjacent to a digit that was one higher or one lower (e.g., a “7” would not be succeeded or preceded by a 6 or 8). The digit span is the longest list recalled. Backward digit span is a measure of working memory, while Forward digit span is a measure of short-term memory. For instance, immediate attention span is commonly tested with the number span [40]. Each correctly repeated list of numbers was worth 1 point, so participants could score a total of 8 or 7 points for digit forward and backward, respectively. We adapted an improved scoring method based on the edit distance [41]; code to compute edit-distance scores with various software is made available at https://osf.io/wdb83/, 8 March 2026.

For the sentence repetition test, participants were asked to repeat 12 sentences from the Serbian version of the BDAE subtest [39] that were presented orally. Each correctly repeated sentence from the BDAE subtest was worth 1 point, so participants could score a total of 12 points on this test. For practice, participants were provided with two sentences and asked to repeat them aloud. Repetition impairment is a core feature of some fluent aphasias (Conduction aphasia and Wernicke’s aphasia) and relatively preserved in some fluent and nonfluent aphasias (e.g., TSA or TMA). However, repetition may be impaired in nonfluent aphasia due to impairments in motor speech disorders such as apraxia of speech [42,43], grammatical processing errors [44,45], or executive dysfunction [46].

Performance on the cognitive tests was scored based on the number of digits/words produced, regardless of phonemic errors (e.g., paraphasias). This approach ensured that reduced performance reflected the number of recalled items rather than language production errors.

### 2.3. Data Analysis

The accuracy of clitic production (percent correct) was recorded for each stimulus and separately for each participant group. Clitic production accuracy was analyzed using an ANCOVA with Group (NFA, FA, HC) and Clitic Type (enclitic, proclitic) as factors, and age, sex, education, and months post-onset as covariates. The associations between different clitic type production and performances on repetition and digit spans were analyzed using mixed-effect logistic regression models. To check for potential outliers from the accuracy data, the mean and standard deviation of percent correct were calculated for each condition of the task, and any participant with three standard deviations above or below the mean was excluded from the analyses. Both ANCOVA and regression were performed using the lme4 package running in the R program (http://www.r-project.org, 8 March 2026). An alpha level of *p* < 0.05 was set for these statistical tests. The adjusted *p*-values were calculated using post hoc estimated marginal means (a.k.a. least-squares means) as implemented in the emmeans package (https://cran.r-project.org/web/packages/lsmeans/index.html, 8 March 2026).

## 3. Results

### 3.1. Patterns of Clitic Production Impairment

Descriptive statistics for clitic production accuracy are summarized in Table 3. Healthy controls showed near-ceiling performance for both enclitics (M = 93.0%, SD = 5.81, range = 77–100) and proclitics (M = 97.0%, SD = 4.47, range = 83–100). Within the aphasia groups, NFA showed the lowest overall performance for both enclitics (M = 43.3%, SD = 22.3, range = 0–83) and proclitics (M = 51.0%, SD = 18.7, range = 8–91). FA exhibited intermediate performance, with higher accuracy for proclitics (M = 70.2%, SD = 15.0, range = 33–91) than for enclitics (M = 57.5%, SD = 18.7, range = 11–80). See Supplemental Material for the data distribution across tasks (Figure S1).

The ANCOVA analysis revealed a significant main effect of Group, *F*(1, 84) = 19.42, *p* < 0.001, indicating overall differences in clitic production accuracy across participant groups (healthy controls > FA > NFA). There was also a significant main effect of Clitic Type, *F*(1, 84) = 6.95, *p* = 0.010, with proclitics being produced more accurately than enclitics. The Group × Clitic Type interaction was not significant, *F*(1, 84) = 0.44, *p* = 0.510, indicating that the effect of clitic type did not differ significantly across groups. Among the covariates, months post-onset significantly predicted clitic production accuracy (*p* = 0.001), whereas age, sex, and education were not significant predictors (*p* > 0.05).

**Figure 1:**
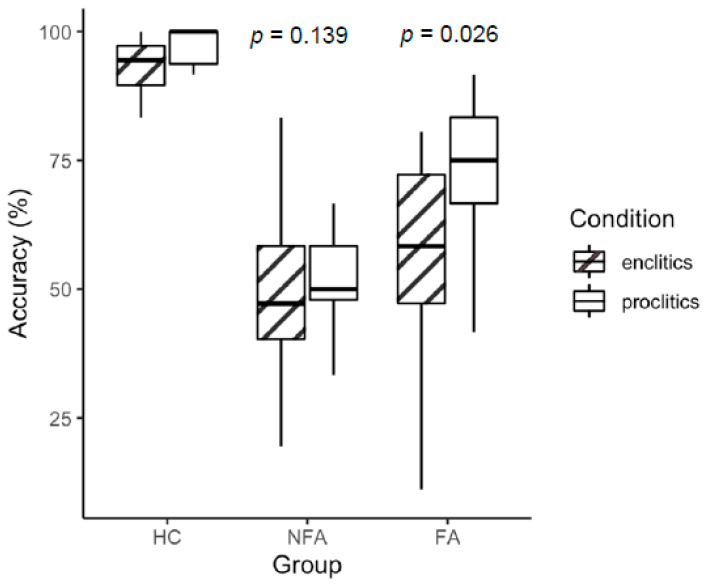
Performance differences across clitic conditions (enclitic and proclitic) for a group of healthy controls (HCs) and participants with nonfluent (NFA) and fluent (FA) aphasia.

Although the interaction was not significant at the omnibus level, planned within-group comparisons were conducted using estimated marginal means from the ANCOVA model, with Tukey adjustment for multiple comparisons. Within-group contrasts revealed a significant proclitic production advantage in FA (*p* = 0.026), whereas no significant clitic-type difference was observed in NFA (*p* = 0.139), after controlling for demographic variables and months post-onset (Figure 1).

### 3.2. Correlations Between Enclitic Production and Cognitive Measures

**Figure 2:**
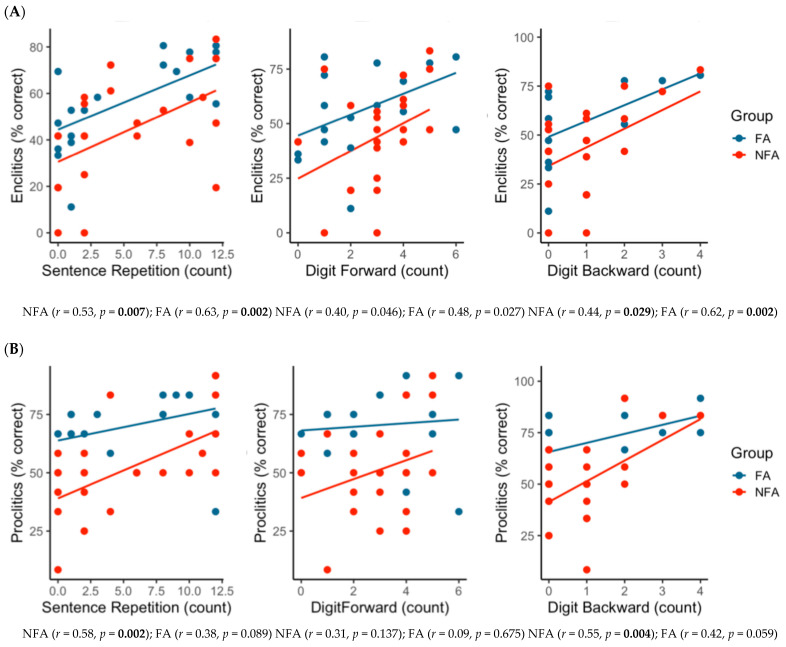
Correlations between the production of enclitic (**A**) and proclitic (**B**) and cognitive measure performances in participants with NFA and FA. Bolded values indicate significant correlations after correction for multiple comparisons.

Associations between enclitic production accuracy and cognitive measures differed by aphasia group (Figure 2A). In the NFA group, enclitic accuracy showed positive relationships with sentence repetition (*r* = 0.53, *p* = 0.007) and digit span backward (*r* = 0.44, *p* = 0.029), indicating that higher verbal short-term and working memory capacity was associated with better enclitic production. The association with digit span forward was weaker (*r* = 0.40, *p* = 0.046) and did not remain significant after correction for multiple comparisons. Similarly, in the FA group, enclitic production was positively associated with sentence repetition (*r* = 0.63, *p* = 0.002) and digit span backward (*r* = 0.62, *p* = 0.002), whereas the relationship with digit span forward was weaker (*r* = 0.48, *p* = 0.027) and did not survive correction for multiple comparisons.

### 3.3. Correlations Between Proclitic Production and Cognitive Measures

A different pattern emerged for proclitic production (Figure 2B). In the NFA group, proclitic accuracy showed significant positive associations with sentence repetition (*r* = 0.58, *p* = 0.002) and digit span backward (*r* = 0.55, *p* = 0.004), whereas the association with digit span forward was not significant (*r* = 0.31, *p* = 0.137). In contrast, in the FA group, proclitic production showed non-significant associations with sentence repetition (*r* = 0.38, *p* = 0.089), digit span forward (*r* = 0.09, *p* = 0.675), and digit span backward (*r* = 0.42, *p* = 0.059). These results suggest that clitic production, both enclitics and proclitics, is more strongly constrained by working memory and sentence-level processing in NFA, whereas FA shows a selective reliance on cognitive processing resources for enclitic production only.

## 4. Discussion

The present study provides the first systematic investigation of clitic production in Serbian-speaking individuals with aphasia, offering novel insights into how different clitic types (enclitic and proclitic) are affected in nonfluent and fluent aphasias and how their production relates to higher-order cognition. Both aphasia groups produced significantly fewer clitics than healthy controls, confirming that clitics constitute a vulnerable grammatical domain in acquired language disorders. Critically, impairment patterns differed across aphasia groups and clitic types, revealing both shared and distinct mechanisms underlying grammatical breakdown in Serbian speakers with aphasia, supporting the use of clitic performance as a potential clinical marker in this population.

### 4.1. Differential Impairments of Enclitic and Proclitic Production Across Aphasia Groups

Individuals with NFA show uniformly reduced performance across clitic types, in contrast to the selective advantage for proclitic production observed in FA. This difference raises interesting issues concerning the processing of the two clitic types. The distinct impairments suggest that enclitics impose greater processing demands than proclitics, likely due to their strict positional constraints, dependence on host structure, and frequent participation in multi-element clitic clusters [11]. In Serbian, *enclitics* must occupy a fixed second-position slot and often co-occur in clusters with auxiliaries or object pronouns, integrating morphosyntactic information related to tense, agreement, and argument structure [10]. These properties likely increase their computational load, making them especially vulnerable when grammatical planning or sequencing mechanisms are compromised. *Proclitics*, by contrast, attach to following words and can appear sentence-initially, reducing linearization and integration demands [11,12,47].

Consistent with this account, the absence of a clitic-type effect in NFA, characterized by general omission of clitics, suggests a more global breakdown of grammatical encoding. Similar patterns were also reported in Italian, French, Spanish, and Finnish agrammatic aphasic speakers [2,3,4,5,6,7,8,9,10,11,12,13,14,15,16,48]. Greek studies also suggest that clitic production is severely impaired in agrammatic aphasia, with more prominent difficulties in the case of post-verbal clitics [26,27,28,49]. The results are also in line with studies in English-speaking individuals with nonfluent aphasia, both post-stroke and primary progressive aphasia, which report omissions of function words in structured elicitation tasks [50,51] and in spontaneous speech [52]. This pattern is consistent with accounts of agrammatism that posit reduced syntactic computation capacity or impaired access to functional categories [44,45].

In contrast, individuals with FA appear to retain partially intact grammatical representations, allowing selective sensitivity to structural complexity to emerge. The proclitic advantage observed in FA suggests partial preservation of grammatical encoding mechanisms, with selective vulnerability for structures requiring precise linearization and integration across multiple representational levels. This pattern parallels findings in Greek and Romance languages, where object clitics and auxiliary combinations are disproportionately impaired relative to other functional elements [22,27]. In Serbian, enclitics may therefore represent a particularly sensitive marker of grammatical complexity rather than a general marker of morphosyntactic deficit.

These findings can be interpreted within the classical distinction between two classical grammatical disturbance patterns: *agrammatism*, typically associated with non-fluent speech and characterized by omission of function words and inflections, and *paragrammatism*, more commonly observed in fluent speech and characterized by word order errors and substitution of grammatical morphemes [53,54,55]. Individuals with NFA showed uniformly reduced performance across clitic types, primarily characterized by clitic omission, which is consistent with an agrammatic speech profile. In contrast, the proclitic advantage observed in FA suggests a different type of grammatical disruption, more consistent with paragrammatic speech. Because enclitics in Serbian must occupy a strict second-position slot and often participate in clitic clusters encoding tense, agreement, and argument structure, their production requires precise sequencing and integration within the sentence. Difficulties with these structures may therefore reflect disruptions in morphosyntactic linearization and integration rather than a complete loss of functional elements. For instance, an individual with FA produced substitutions and circumlocutory constructions rather than omissions of clitics. Instead of the target sentence ‘Učenici sede *na* drvenim stolicama’ (‘Students sit on wooden chairs’), the individual with FA produced ‘Pa *u* stolici, *u* stolicama su bili, sedeli su, oni tamo sede’ (“Well, in the chair, in the chairs they were, they were sitting, they are sitting there.”). Future studies should examine item-level responses to further evaluate this classical grammatical distinction.

### 4.2. Role of Verbal and Working Memory in Clitic Production Across Aphasia Groups

The correlational analyses further clarify the cognitive mechanisms supporting clitic production. Across groups, clitic accuracy, especially for enclitics, was associated with sentence repetition and working memory, with the strongest and most consistent relationships observed for backward digit span. This pattern indicates that clitic production relies not only on morphosyntactic knowledge but also on domain-general working memory resources that support maintenance and manipulation of linguistic material.

In NFA, both enclitic and proclitic production correlated with sentence repetition and backward digit span, a classic measure of working memory manipulation. From a processing perspective, NFA has been characterized by reduced processing speed, limited working memory capacity, and impaired sequencing of morphosyntactic elements [36,56,57]. These deficits may overcome any distinctions between simpler and more complex clitic types, resulting in uniformly low accuracy. In NFA, both enclitic and proclitic production depended on these resources, suggesting that limited working memory capacity broadly constrains grammatical output. These findings align with models proposing that grammatical encoding draws on domain-general working memory resources, especially under conditions of increased structural complexity [44,46]. Enclitics, which require maintaining agreement, case, and positional constraints, may place especially high demands on working memory, explaining their strong association with backward digit span. The fact that similar relationships were observed for proclitics in NFA further supports the view that resource limitations override structural differences when grammatical impairment is severe.

In FA, by contrast, a more selective pattern emerged. Here, enclitic production, but not proclitic production, was significantly associated with sentence repetition and backward digit span. This suggests that FA preserves some automatic or lexically driven aspects of grammatical production, while difficulties arise specifically for structures that require higher-order integration across syntactic and memory domains [58,59]. Enclitics, which often participate in clitic clusters and require precise ordering, appear to tax these resources more strongly than proclitics.

The differential involvement of digit span forward versus backward further supports a distinction between passive storage and active manipulation [60,61]. Across groups, backward digit span showed the strongest and most consistent associations with clitic production, reinforcing the idea that working memory manipulation, rather than simple short-term storage, is critical for producing morphosyntactic complex elements (in line with studies on elderly individuals [60]). The Serbian clitic system, with its rigid ordering constraints and rich morphology, therefore, offers a powerful tool for probing how grammatical complexity and cognitive resources jointly shape aphasic performance.

Taken together, the results point to both shared and distinct mechanisms underlying clitic impairment in NFA and FA. Shared features include overall reduced accuracy relative to healthy controls and a strong link between clitic production and cognitive abilities. These commonalities suggest that clitic production taps into core mechanisms of grammatical encoding that are vulnerable across aphasia types. At the same time, the presence of a proclitic advantage only in FA indicates qualitative differences in how linguistic and cognitive resources are deployed. In FA, grammatical representations may remain partially intact but become selectively vulnerable when processing demands increase. In NFA, by contrast, more pervasive deficits in morphosyntactic encoding and sequencing, as well as working memory, may obscure distinctions between clitic subtypes.

### 4.3. Clinical Implications of Clitic Production in Aphasia

The near-ceiling performance of healthy controls supports the sensitivity of the SCP test and suggests that the observed deficits are not attributable to task difficulty or lexical frequency differences, which were controlled across clitic types. At the same time, the richness of the Serbian clitic system allows finer-grained distinction between enclitic and proclitic processing in aphasia types that have been difficult to observe in other languages. Clinically, the findings highlight the diagnostic value of clitic production tasks in Serbian. These findings have important implications for treatment, particularly given that several morphosyntactic intervention approaches have primarily focused on training inflectional morphemes (see 32 for review). For example, Morphosemantic Treatment [62], originally developed in English, has been shown to improve verb tense inflection in Persian-speaking individuals with nonfluent aphasia [63,64]. Similarly, ACTION therapy [65], designed to treat verb production deficits in Dutch, has demonstrated cross-linguistic effectiveness in improving verb inflection and retrieval in languages such as Persian and Italian [66]. These findings suggest that therapies targeting grammatical encoding mechanisms may generalize across languages. The present results extend this literature by identifying clitic production as a potential therapeutic target in Serbian, particularly given its sensitivity to grammatical encoding demands. Moreover, the strong association between clitic production and working memory observed in this study suggests that interventions addressing working memory may support morphosyntactic recovery. Although relatively few studies have investigated the impact of cognitive factors on response to post-stroke aphasia treatment, and findings have been mixed [61], evidence from developmental language disorders indicates that training working memory can transfer to morphosyntactic skills [67]. However, this possibility has yet to be tested in individuals with aphasia.

The results support models in which clitic production emerges from interactions between grammatical processing and domain-general cognitive resources. Serbian, with its rich and strictly ordered clitic system, provides a powerful test case for examining these interactions. The present findings contribute cross-linguistic evidence that grammatical vulnerability in aphasia cannot be explained solely by morphosyntactic complexity or frequency but must also account for processing demands imposed by memory and sequencing constraints.

### 4.4. Limitations

Several limitations should be acknowledged. First, although the sample size is comparable to prior aphasia studies, subgroup sizes, particularly within FA, remain modest, limiting finer-grained comparisons among specific aphasia subtypes. Second, lesion location and extent were not incorporated into the present analyses; future work integrating neuroimaging could clarify the neural substrates underlying clitic deficits. Third, the SCP test focuses on elicited production; examining spontaneous speech and comprehension would provide a more comprehensive picture of clitic processing in aphasia. Differences in clitic production in aphasia may reflect not only variation in the grammatical properties of clitics across languages [68,69], but also differences in the methodological approaches (e.g., different tasks) adopted across studies [70]. Additionally, from a pragmatic perspective, the tasks involved simple everyday situations and did not require complex inferencing, discourse integration, or extended narrative production. The primary demand was grammatical encoding rather than pragmatic interpretation. However, as in most elicited production tasks, some degree of contextual interpretation and sentence planning was necessary. Therefore, it is possible that individuals with broader cognitive–communication impairments could experience additional difficulty related to working memory rather than clitic production per se. Although the Test demonstrates strong psychometric properties, including internal consistency, test–retest reliability, inter-rater reliability, and intra-rater reliability, stimulus characteristics as well as participant variables (e.g., education level or regional variation within the Serbian language area) may still influence performance in some cases. For this reason, a detailed qualitative analysis of item-level responses and error patterns will be an important next step in future research. Lastly, although participants were clinically judged to hear speech at normal conversational intensity and practice items were used to verify comprehension and audibility in a quiet testing environment, objective audiological screening was not conducted. Future studies should include formal hearing screening and examine clitic processing across tasks in a larger cohort.

## 5. Conclusions

This study demonstrates that clitic production of the present language, Serbian, is differentially impaired across aphasia types and related to working memory and sentence-level processing. While both NFA and FA show reduced enclitic production, only FA exhibits a selective proclitic advantage, indicating partial preservation of grammatical mechanisms. Enclitics emerge as especially sensitive markers of morphosyntactic and cognitive load, drawing heavily on working memory resources. These findings underscore the value of Serbian clitic production as a window into the interaction between grammar and cognition in aphasia and provide a foundation for future cross-linguistic and clinically oriented research.

## Figures and Tables

**Table 1:** Demographics, cognitive, and speech-language scores for individuals with NFA and FA and a group of healthy controls (HCs). Group differences (*p* < 0.05; one-way ANOVA and Tukey HSD Test).

	HC	NFA	FA	*p*-Values
*N*	54	25	21	
Age (number of years)	52.9 (17.9)	61.5 (8.2)	57.4 (11.8)	0.074
Education (number of years)	14.1 (2.0)	13.8 (2.7)	13.2 (2.4)	0.278
Sex, n (%) female	33 (61%)	9 (43%)	5 (16%)	0.003
Post-onset of stroke (months)	---	10.4 (5.0)	11 (5.9)	0.728
Severity of aphasia	---	303 (79.4)	332 (111.0)	0.304
Digit Span Forwards	7.0 (1.2) ^NFA,FA^	2.7 (1.8)	2.9 (1.4)	<0.001
Digit Span Backwards	5.3 (1.3) ^NFA,FA^	1.1 (1.4)	0.9 (1.0)	<0.001
Digit Span (total count)	12.3 (2.2) ^NFA,FA^	3.7 (2.8)	3.8 (2.1)	<0.001
Sentence Repetition	11.9 (0.3) ^NFA,FA^	5.6 (4.9)	5.0 (4.5)	<0.001
The Boston Diagnostic Aphasia Examination (BDAE) tests				
Fluency	---	8.16 (2.81)	13.9 (2.19)	<0.001
Word discrimination (max 72)	---	69.7 (3.66)	61.2 (15.8)	0.012
Word-picture matching (max 10)	---	8.44 (2.48)	8.81 (1.94)	0.582
Responsive naming (max 30)	---	19.2 (7.16)	18.7 (6.51)	0.797
Visual Confrontation naming (max 105)	---	64 (19.2)	57.4 (21.1)	0.269
Animal naming (60 s)	---	1.64 (1.41)	4.05 (1.88)	<0.001
Cookie Theft Picture (number of words)	---	9 (3.19)	17.2 (5.85)	<0.001

**Table 2:** Serbian Clitic Production (SCP) test example items, including discourse scenarios, eliciting questions, and expected responses.

	Scenarios	Question	Response
Enclitics (*N* = 18, 6 each)			
Reflexive pronoun (PRON): yourself/se	Sara se razbolela. Ona nije otišla u školu.	Zašto Sara nije otišla u školu?	Ona/Sara se _PRON_ razbolela./Razbolela se _PRON_.
Auxiliary verb (AUX): want/će	Glumac će biti veoma zadovoljan. Dobiće ulogu u novom filmu.	Zašto je glumac srećan?	Zato što će _AUX_ dobiti novu ulogu./Glumac će _AUX_ dobiti novu ulogu.
Clitic Pronoun-Auxiliary (PRON-AUX): It is/ga je	Mačka je popila mleko. Mleka nema više.	Zašto nema više mleka?	Popila ga je _PRON-AUX_ mačka./Mleka nema više zato što ga je _PRON-AUX_ popila mačka.
Proclitics (*N* = 12, 6 each)			
Prepositions (PREP): from/iz	Marija i Marko se odmaraju. Oni su skoro došli iz škole.	Odakle su došli Marija i Marko?	Marija i Marko su došli iz _PREP_ škole.
Conjunctions (CONJ): or/ili	Nina ide na rođendan kod drugarice. Ona će obući bele pantalone ili crvenu haljinu.	Šta će Nina obući?	Bele pantalone ili _CONJ_ crvenu haljinu.

**Table 3:** Mean and standard deviations, and range of clitic production accuracy (percent correct) across clitic types for a group of healthy controls (HCs) and participants with nonfluent (NFA) and fluent (FA) aphasia.

Group	Clitic Types	Mean (SD)	Range
HC	enclitic	93.0 (5.81)	77–100
NFA	enclitic	43.3 (22.3)	0–83
FA	enclitic	57.5 (18.7)	11–80
HC	proclitic	97.0 (4.47)	83–100
NFA	proclitic	51.0 (18.7)	8–91
FA	proclitic	70.2 (15.0)	33–91

## Data Availability

Anonymized language and cognitive data are available upon request from the authors (mvukovic@fasper.bg.ac.rs; slukic@fsu.edu) due to privacy and ethical restrictions.

## Supplementary Materials

The following supporting information can be downloaded at https://www.mdpi.com/article/10.3390/brainsci16030324/s1, Figure S1: Distribution of data on enclitic or proclitic (percent correct), digit spans, and sentence repetition (row values) across healthy controls (HCs) and participants with nonfluent (NFA) and fluent (FA) aphasia.

## Appendix A

**No**	**Clitic Responses**
	Enclitics
1	Mačka je popila mleko. Mleka nema više. Zašto nema više mleka? (ciljani odgovor: ga, je–Mleka nema više zato što *ga je* popila mačka.)
2	Kuvarica je nezadovoljna. Kuvarici je zagoreo ručak. Zašto je kuvarica nezadovoljna? (ciljani odgovor: joj, je –Kuvarica je nezadovoljna zato što *joj je* zagoreo ručak.)
3	Mačka je ogrebala dečaka. Dečak place. Zašto dečak place? (ciljani odgovor: ga, je–Dečak plače zato što *ga je* mačka ogrebala.)
4	Vetar je polomio Markov kišobran. Marko je mokar. Zašto je Marko mokar? (ciljani odgovor: mu, je– Zato što *mu je* polomljen kišobran./Zato što *mu je* vetar polomio kišobran.)
5	Veliki talasi su zapljuskivali obalu. Obali se nije moglo prići. Zašto se nije moglo prići obali? (ciljani odgovor: su, je–Obali se nije moglo prići zato što *su je* zapljuskivali veliki talasi.)
6	Policajac je kaznio vozača. Vozač je besan. Zašto je vozač besan? (ciljani odgovor: ga, je–Vozač je besan zato što *ga je* kaznio policajac.)
7	Marko je oprao auto. Njegov auto se sija. Zašto se Markov auto sija? (ciljani odgovor: ga, je–Auto se sija zato što *ga je* Marko oprao.)
8	Deca su pojela sve bombone. Bombona više nema u kući. Zašto više nema bombona. (ciljani odgovor: su, ih–Bombona više nema zato što *su ih* deca pojela.)
9	Lepa devojka je namignula mladiću. Mladić je zadovoljan. Zašto je mladić zadovoljan? (ciljani odgovor: mu, je–Mladić je zadovoljan zato što *mu je* namignula lepa devojka.)
10	Mama je oprala Sarinu jaknu. Sare ne može da ide napolje. Zašto Sara ne može da ide napolje? (ciljani odgovor: joj, je–Sara ne može da ide napolje zato što *joj je* mama oprala jaknu.)
11	Petru je jutros pozlilo. Petar nije došao na posao. Zašto Petar nije došao na posao? (ciljani odgovor: mu, je–Petar nije došao na posao zato što *mu je* pozlilo.)
12	Ana se razbolela. Ona nije otišla u školu. Zašto Ana nije otišla u školu? (ciljani odgovor: se–Ana nije otišla u školu zato što *se* razbolela.)
13	Glumac je veoma srećan. Dobiće ulogu u novom filmu. Zašto je glumac srećan? (ciljani odgovor: će–Glumac je srećan zato što *će* dobiti ulogu u novom filmu.)
14	Prodavac nije Milutinu vratio kusur. Milutin je besan. Zašto je Milutin besan? (ciljani odgovor: mu–Milutin je besan zato što *mu* prodavac nije vratio kusur.)
15	Majina kosa je neuredna. Ona je uzela češalj. Šta će Maja uraditi češljem? (ciljani odgovor: će se–Maja *će se* očešljati/počešljaće *se*/očešljaće *se*)
16	Jovan i Miloš sutra putuju na more. Njima ce mama spakovati stvari. Šta će mama uraditi za Jovana i Miloša? (ciljani odgovor: će, im–Mama *će im* spakovati stvari./Spakovaće *im* stvari./Spremiće im stvari.)
17	Luku i Nokolu bole kolena. Oni ne mogu da trče. Zašto Luka i Nikola ne mogu da trče? (ciljani odgovor: ih–Luka i Nikola ne mogu da trče zato što *ih* bole kolena.)
18	Katarina nije mogla da spava. Katarinu je boleo zub. Zašto katarina nije mogla da spava? (ciljani odgovor: ju, je–Katarina nije mogla da spava zato što *ju je* boleo zub.)
	Proclitics
19	Mama je rekla Jeleni: Jelena dođi! Šta je mama rekla Jeleni? (ciljani odgovor: da, Mama je rekla Jeleni *da* dođe.)
20	Jovana ne ide na more. Jovana je tužna. Zašto je Jovana tužna? (ciljani odgovor: jer, zato što–Jovana je tužna *jer* ne ide na more.)
21	Sara je juče kupila olovku. Danas je kupila knjigu. Šta je sve Sara kupila? (ciljani odgovor: i–Sara je kupila olovku *i* knjigu.)
22	Milena ima crni šešir. Ivana ima plavi šešir. Koje je boje Milenin, a koje Ivanin šešir? (ciljani odgovor: a–Milenin šešir je crne, *a* Ivanin plave boje.)
23	Učiteljica je rekla da će dobiti minus svaki učenik koji ne donese bojice ili flomastere. Saša je dobio minus. Zašto je Saša dobio minus? (ciljani odgovor: ni–Saša je dobio minus zato što nije doneo (*ni*) bojice *ni* flomastere.)
24	Nina ide na rođendan kod drugarice. Ona će obući bele pantalone ili crvenu haljinu. Šta će Nina obući? (ciljani odgovor: ili–Nina će obući bele pantalone *ili* crvenu haljinu.)
25	Losos je vrsta ribe. Njegovo stanište je Atlantski ocean. Gde živi losos? (ciljani odgovor: u–Losos živi *u* Atlantskom okeanu, *u* okeanu, *u* moru.)
26	Učenici su u učionici. Njihove stolice su drvene. Na čemu sede učenici? (ciljani odgovor: na-Učenici sede *na* drvenim stolicama.)
27	Golub je u sobi. Prozor je otvoren. Kuda je golub uleteo u sobu? (ciljani odgovor: kroz–Golub je uleteo u sobu *kroz* prozor.)
28	Marija i Marko su bili u školi. Došli su kuci i odmaraju se. Odakle su došli Marija i Marko? (ciljani odgovor: iz–Marija i Marko su došli *iz* škole).
29	Nikola je otišao na aerodrom. Odatle je otputovao u Moskvu. Odakle je poleteo avion? (ciljani odgovor: sa–Avion je poleteo *sa* aerodroma.)
30	Milica je otišla da izvadi zub. Gde je otišla Milica? (ciljani odgovor: kod–Milica je otišla *kod* zubara/stomatologa)
