# Supplementary material for: The Production of Clitics in Serbian Speakers with Stroke Aphasia
Source: Brain Sci. 2026 Mar 19;16(3):324. doi: 10.3390/brainsci16030324 (PMC13023991; doi:10.3390/brainsci16030324)
Supplement: Supplementary file 1 [file brainsci-16-00324-s001.zip › brainsci-4173869-supplementary.pdf]

Supplementary Material

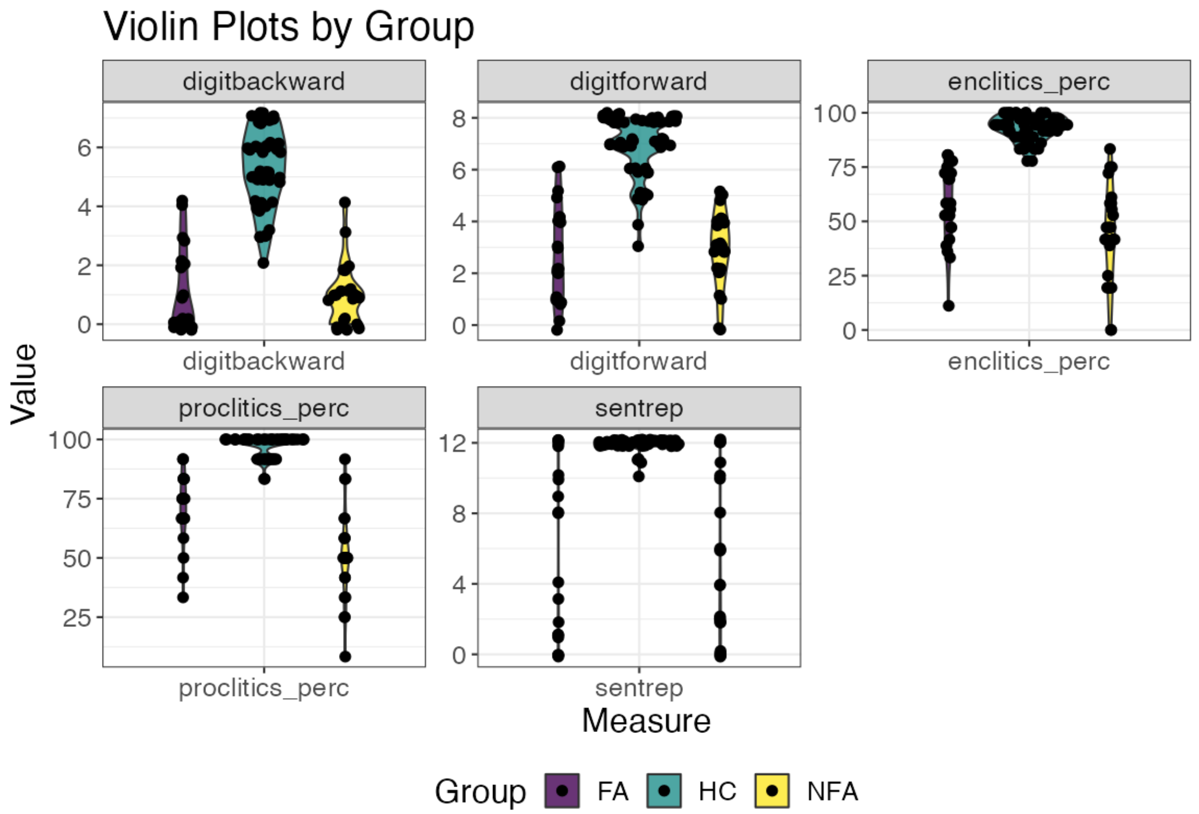

Figure S1. Distribution of data on enclitic or proclitic (percent correct), digit spans, and sentence repetition (row values) across healthy controls (HC) and participants with nonfluent (NFA) and fluent (FA) aphasia.
